# Acute Salivary Biomarker, Cognitive, and Tennis Performance Responses to Tennis-Specific Exercise Versus HIIT: A Randomized Crossover Study

**DOI:** 10.3390/life16091565

**Published:** 2026-09-18

**Authors:** Harun Genç, Mustafa Türkmen, Mehmet Şerif Ökmen, İsmail Polatcan, İsmail Kirkpinar, Muhammed Şahin, Ramazan Baltaci, Hasan Şahin, Furkan İdgö, Alperen Erkin

**Affiliations:** 1 Faculty of Sports Sciences, Bingöl University, Bingöl 12000, Türkiye; 2 Faculty of Sports Sciences, Mardin Artuklu University, Mardin 47100, Türkiye; mustafaturkmen@artuklu.edu.tr (M.T.); mserifokmen@gmail.com (M.Ş.Ö.); ismailpolatcan@artuklu.edu.tr (İ.P.); ismailkirkpinar@artuklu.edu.tr (İ.K.); muhammedsahin@artuklu.edu.tr (M.Ş.); furkan.idgo61@gmail.com (F.İ.); 3 Faculty of Sports Sciences, Gaziantep University, Gaziantep 27310, Türkiye; ramazanbaltaci2676@gmail.com; 4 Faculty of Sports Sciences, Turgut Özal University, Malatya 44210, Türkiye; hasansahin0077@gmail.com; 5 Faculty of Sports Sciences, Ondokuz Mayıs University, Samsun 55200, Türkiye; alperenerkin7@gmail.com

**Keywords:** tennis, high-intensity interval training, salivary biomarkers, salivary creatine kinase, cognitive performance

## Abstract

Tennis requires the integration of physiological, technical, and cognitive abilities during intermittent high-intensity exercise; however, it remains unclear whether tennis-specific exercise induces different acute responses from duration-matched high-intensity interval training (HIIT). This randomized crossover study compared the acute effects of a tennis-specific exercise protocol (TSEP) and duration-matched HIIT on salivary biomarkers, working memory-related cognitive performance, and tennis-specific performance in 12 amateur competitive male tennis players (22.1 ± 1.6 years). Participants completed both protocols separated by a 7-day washout period. Salivary alpha-amylase (sAA) and secretory immunoglobulin A (sIgA) were assessed before, immediately after, and 30 min after exercise, whereas salivary creatine kinase (CK) was assessed before and 24 h after exercise. Tennis-specific accuracy, auditory 2-back target detection accuracy, and separated and combined dual-task performance were evaluated before and immediately after each protocol. Dual-task costs (DTCs) were also calculated separately for the motor and cognitive components. Both protocols produced significant time-dependent changes in sAA and sIgA (both *p* < 0.001), without significant condition or Condition × Time interaction effects. Salivary CK increased following both protocols, with a significantly greater Pre-to-Post-24 h increase following HIIT (Condition × Time, *p* = 0.032). The binomial generalized linear mixed-effects models showed significant overall time effects for single-task forehand and backhand accuracy and single-task 2-back target detection accuracy, whereas the time effect for serve accuracy was not statistically significant. However, none of the single-task performance outcomes showed a significant Condition × Time interaction. Among the separated dual-task outcomes, significant overall time effects were observed for backhand and cognitive accuracy, whereas forehand and serve accuracy showed no significant time effects. Combined dual-task accuracy also showed a significant overall time effect. None of the separated or combined dual-task outcomes showed a significant Condition × Time interaction. Relative motor and cognitive DTC outcomes were summarized descriptively and treated as exploratory because of non-normality, denominator instability, and one undefined value. Overall, both exercise protocols were followed by changes in selected salivary biomarkers and declines in selected technical and cognitive performance outcomes, but the analyses provided insufficient evidence that the magnitude of technical, cognitive, or dual-task performance change differed between TSEP and HIIT. In contrast, HIIT elicited a greater delayed salivary CK response. A multidimensional assessment combining physiological, biochemical, technical, cognitive, perceptual, and external-load measures may therefore provide a more comprehensive approach to monitoring acute fatigue in tennis.

## 1. Introduction

Tennis is an intermittent sport characterized by successive bouts of high-intensity, short-duration activity interspersed with lower-intensity recovery periods, during which physical and cognitive demands are simultaneously prominent [1,2]. During match play, athletes must effectively utilize physical capacities such as speed, agility, explosive power, and anaerobic capacity, as well as higher-order cognitive processes including attention, decision-making, reaction time, and cognitive flexibility [3]. This multidimensional performance profile necessitates a holistic evaluation of the acute effects of training and competition loads on athletes.

In recent years, salivary biomarkers have been widely used as a non-invasive method for monitoring physiological stress and recovery status in athletes [4]. In particular, salivary α-amylase (sAA) and secretory immunoglobulin A (sIgA) are commonly used biomarkers for assessing sympathetic nervous system responses and mucosal immune function, respectively. These biomarkers provide valuable insight into acute exercise-induced physiological stress and recovery [4,5]. Although high-intensity exercise has been shown to induce acute changes in these biomarkers, studies examining biomarker responses during sport-specific loading in tennis remain limited.

High-intensity interval training (HIIT) is widely recognized as an effective training method for enhancing tennis performance [6]. HIIT protocols combine short bouts of maximal or supramaximal exercise with recovery intervals, thereby targeting both aerobic and anaerobic energy systems [7]. Although HIIT is known to markedly influence acute physiological stress responses [4,7], comparing these responses with those elicited by sport-specific loading, such as tennis-specific exercise, may provide valuable information for optimizing tennis-specific training programs.

Moreover, the effects of intense exercise on cognitive performance components, including attention, executive functions, and reaction time, have been increasingly investigated in recent years, and their relationship with sports performance has been examined across different sporting disciplines [8]. Changes in attention, reaction time, and decision-making following exercise are of particular importance in sports such as tennis, where rapid decision-making is essential and may directly influence performance [3]. Working memory, a fundamental component of cognitive performance, plays an important role in the short-term processing and updating of environmental information, as well as in planning appropriate motor responses. Similarly, cognitive–motor performance (dual-task performance), which refers to the simultaneous execution of cognitive and motor tasks, is critical in open-skill sports such as tennis. In these sports, athletes must perceive environmental stimuli, allocate attention across multiple cues, and make appropriate technical and tactical decisions. Findings from systematic reviews indicate that dual-task paradigms can acutely influence both cognitive and motor performance in athletes; however, research in this area remains limited [9].

Evaluating the effects of acute exercise-induced physiological stress responses on cognitive performance is important for understanding the physiological and neurocognitive mechanisms that jointly determine athletic performance [8]. Within this context, previous research has shown that sport-specific HIIT may impose a greater physiological load than non-playing HIIT and may affect tennis performance, particularly stroke accuracy [10]. However, these studies have predominantly focused on physiological and performance outcomes. Studies comparing the acute effects of tennis-specific exercise and HIIT protocols on salivary biomarkers, cognitive performance, cognitive–motor performance, and tennis performance within the same group of athletes remain scarce. Therefore, the aim of the present study was to compare acute responses in salivary biomarkers, tennis performance, cognitive performance, and cognitive–motor performance following a tennis-specific exercise protocol (TSEP) and a HIIT protocol in tennis players using a crossover design.

Accordingly, it was hypothesized that the HIIT protocol would elicit a greater physiological stress response and lead to more pronounced acute changes in tennis-specific technical performance. It was further hypothesized that the two exercise protocols would produce different acute responses in cognitive and cognitive–motor performance.

## 2. Materials and Methods

### 2.1. Sample Size Calculation

An a priori sample-size calculation was performed using G*Power software (version 3.1.9.7; Heinrich Heine University, Düsseldorf, Germany). The calculation used the “ANOVA: Repeated measures, within factors” procedure and assumed a large effect size (f = 0.40), an α level of 0.05, statistical power of 0.80, three repeated measurements, a correlation of 0.50 among repeated measurements, and a nonsphericity correction of ε = 1.00. The large effect-size assumption was based on Cohen’s conventional benchmark [11] rather than on pilot data or an estimate derived from a specific previous study. The analysis indicated that 12 participants were required, corresponding to an actual power of 0.816.

No single outcome was prospectively designated as the primary outcome, and the calculation represented a general within-participant effect across three repeated measurements rather than the Condition × Time interaction subsequently examined in the mixed-effects models. Therefore, the study was not specifically powered for the Condition × Time interaction or for each individual biomarker, cognitive, and tennis-specific performance outcome. Accordingly, these analyses should be regarded as exploratory.

### 2.2. Participants

The study included 12 healthy amateur male tennis players with competitive experience, aged 19–25 years. Participants had at least two years of tennis experience, trained at least three times per week, had sustained no musculoskeletal injuries during the previous six months, and had no known cardiovascular, neurological, or metabolic disorders that could affect exercise performance or compromise participant safety.

Table 1 presents the descriptive characteristics of the participants. The participants (*n* = 12) had a mean age of 22.08 ± 1.56 years, a mean height of 174.16 ± 4.62 cm, a mean body mass of 72.12 ± 9.16 kg, and a mean body mass index (BMI) of 23.72 ± 2.34 kg/m^2^.

### 2.3. Inclusion and Exclusion Criteria

#### 2.3.1. Inclusion Criteria

Participants were eligible for inclusion if they met the following criteria: (i) male, aged between 18 and 25 years; (ii) had at least two years of tennis experience and had participated in regular tennis training at least three times per week during the preceding six months; (iii) were free from any chronic disease or medical condition that would contraindicate high-intensity exercise; (iv) had no history of musculoskeletal injury within the previous six months; and (v) voluntarily agreed to participate by providing written informed consent.

#### 2.3.2. Exclusion Criteria

Participants were excluded if they: (i) had any musculoskeletal injury or disorder, or any cardiovascular, neurological, or metabolic disorder that could affect exercise performance or compromise participant safety; (ii) were taking medications known to influence physical performance, cognitive function, or physiological responses; (iii) were unable to comply with the study protocol; or (iv) withdrew their informed consent at any stage of the study.

This study employed a randomized crossover design to compare the acute effects of a tennis-specific exercise protocol (TSEP) and a duration-matched high-intensity interval training (HIIT) protocol on salivary biomarkers, cognitive performance, cognitive–motor performance, and tennis performance in amateur male tennis players with competitive experience. Following baseline assessments, participants were randomly assigned in a 1:1 ratio to one of two intervention sequences (*n* = 6 per sequence) using a computer-generated random-number list. The allocation sequence was generated by an independent researcher who was not involved in participant recruitment, outcome assessment, or protocol administration. To ensure allocation concealment, sequence assignments were placed in sequentially numbered, opaque, sealed envelopes and were opened only after participant enrolment and completion of the baseline assessments. Sequence A completed the HIIT protocol followed by TSEP, whereas Sequence B completed TSEP followed by HIIT. The two experimental sessions were separated by a 7-day washout period to minimize potential carryover effects. All participants completed both exercise conditions without withdrawal; therefore, data from all 12 participants were included in the final analyses. Experimental testing was conducted over approximately two weeks under standardized environmental conditions. To minimize potential confounding effects between sessions, participants were instructed to refrain from strenuous exercise, avoid alcohol and caffeine, and maintain their usual dietary and sleep habits during the 24 h preceding each experimental session. Compliance with these instructions was confirmed verbally before each session. Salivary biomarkers, cognitive performance, cognitive–motor performance, and tennis performance were assessed at predetermined time points before and after each exercise protocol. The TSEP and HIIT protocols were matched for total duration (40 min) but differed in exercise characteristics and prescribed intensity distribution (Table 2). Period and sequence were included as fixed effects in the mixed-effects models to account for potential influences related to the crossover design.

### 2.4. Tennis-Specific Exercise Protocol

The TSEP was designed to replicate the physiological and movement demands of competitive tennis while maintaining a standardized total duration of 40 min. The protocol consisted of a tennis-specific warm-up, two identical 6 min exercise blocks, a 1 min seated passive recovery and 3 min active recovery between the two exercise blocks, a 1 min seated passive recovery following the second exercise block, and a 10 min cool-down phase.

Following 5 min of low-intensity running (30% HRR) and 5 min of tennis-specific dynamic stretching, participants completed a 3 min tennis-specific warm-up consisting of short rallies and serve-return drills (70% HRR), resulting in a 13 min warm-up period.

The main protocol comprised two identical 6 min exercise blocks. Each block consisted of 3 min of baseline groundstrokes incorporating forehand and backhand strokes, change-of-direction movements, and topspin drills (80% HRR), followed by 2 min of net play incorporating slice and topspin combinations (90% HRR), and 1 min of high-intensity serve practice combined with rapid-reaction drills (95% HRR). The drills were time- and HRR-based rather than repetition- or distance-based; therefore, no fixed number of stroke repetitions or predetermined movement distance was prescribed within the exercise blocks.

The first exercise block was followed by a 1 min seated passive recovery and a 3 min active recovery consisting of mini-rally play at 40% HRR. Following the second exercise block, participants completed a 1 min seated passive recovery.

The protocol concluded with a 10 min cool-down consisting of 5 min of low-intensity jogging at 30% HRR and 5 min of static stretching.

The total duration of the TSEP was 40 min.

### 2.5. High-Intensity Interval Training Protocol

The HIIT protocol was designed as a duration-matched high-intensity exercise condition with a standardized total duration of 40 min. The TSEP and HIIT protocols were matched for total exercise duration but differed in exercise modality and prescribed intensity distribution. The HIIT protocol consisted of a standardized warm-up, two identical 6 min interval exercise blocks, a 1 min seated passive recovery and 3 min active recovery between the two exercise blocks, a 1 min seated passive recovery following the second exercise block, and a 10 min cool-down/recovery phase.

Following 5 min of low-intensity running (30% HRR), 5 min of dynamic stretching, and 3 min of light running (50% HRR), participants completed a 13 min warm-up period.

The main protocol comprised two identical 6 min interval exercise blocks. Each block consisted of 3 min of continuous running at 90% HRR, followed by 2 min at 95% HRR and 1 min at maximal intensity (100% HRR). All running intervals were performed continuously around the perimeter of the indoor tennis court. The HIIT protocol was prescribed based on exercise duration and individualized HRR targets rather than a fixed running distance; therefore, no predetermined running distance was specified.

The first interval exercise block was followed by a 1 min seated passive recovery and a 3 min active recovery consisting of low-intensity jogging at 30% HRR. Following the second interval exercise block, participants completed a 1 min seated passive recovery.

The protocol concluded with a 10 min cool-down/recovery period consisting of 5 min of passive recovery and 5 min of low-intensity jogging at 30% HRR.

The total duration of the HIIT protocol was 40 min.

### 2.6. Experimental Procedures

All experimental sessions were conducted on the same indoor hard tennis court under standardized environmental conditions, with an ambient temperature of approximately 21.3 °C and relative humidity of 42.7%, to minimize external influences on physiological and performance responses. Testing sessions were scheduled at the same time of day (±1 h) for each participant to reduce the potential effects of circadian variation. All participants used the same personal racket, string setup, and tennis shoes in both experimental sessions. The same brand and type of tennis balls were used throughout all testing sessions, and the court surface, target locations, and overall court setup were kept identical across the TSEP and HIIT conditions.

Participants were instructed to refrain from strenuous physical activity for 24 h before each experimental session and to avoid alcohol and caffeine consumption for at least 12 h before testing. They were also asked to maintain their usual dietary and sleep habits and to consume their last meal at least 2 h before each session. Water intake was permitted until 30 min before testing, after which participants were instructed to refrain from fluid consumption until the experimental procedures began. During the 7-day washout period, participants were instructed to maintain their habitual physical activity and avoid unusually strenuous or intensive training that could influence responses during the subsequent session.

Upon arrival at the testing venue, participants remained seated quietly for 10 min, during which resting heart rate (HRrest) was determined. Immediately following this 10 min seated resting period, the Pre saliva sample was collected, followed by the remaining pre-exercise assessments (tennis accuracy, single-task 2-back, and dual-task assessments). Exercise intensity for both protocols was individually prescribed using the Karvonen heart rate reserve (HRR) method. Maximum heart rate (HRmax) was estimated using the age-predicted equation [12]:

HR max = 208 − (0.7 × age)

Target heart rate (THR) for each exercise intensity was subsequently calculated using the Karvonen equation:

THR = [(HR max − HR rest) × Exercise Intensity] + HR rest

Heart rate was monitored continuously throughout both exercise protocols using a Polar H10 heart rate sensor (Polar Electro Oy, Kempele, Finland) to guide exercise intensity in real time. However, the complete time-stamped HR time series was not retained as part of the study dataset. The HR data retained for subsequent analysis consisted of session-level mean and peak HR values, from which the corresponding percentages of HRR were calculated using the Karvonen method. Therefore, retrospective calculation of the time spent within predefined HRR zones was not possible. The observed mean and peak heart rate responses during both exercise protocols are reported in Supplementary Table S4.

The two exercise protocols were performed using a randomized two-period crossover design and were separated by a 7-day washout period. The washout period was implemented to minimize potential residual effects and permit physiological recovery between experimental sessions. Potential period, sequence, and residual carryover effects were evaluated as described in the Statistical Analysis section.

Outcome measures were collected at predefined time points according to the variable assessed. Salivary sAA activity and sIgA concentration were assessed at Pre, Post, and Post-30 min. Salivary CK activity was assessed at Pre and Post-24 h. Cognitive performance, assessed using the 2-back and dual-task tests, and tennis-specific performance, assessed using forehand, backhand, and serve accuracy tests, were evaluated at Pre and Post.

Following completion of each exercise protocol, post-exercise assessments were conducted in a standardized sequence. Saliva collection began immediately after exercise cessation and was completed within approximately 2 min. Tennis accuracy assessments were subsequently performed and required approximately 5 min in total, followed by the single-task auditory 2-back assessment (approximately 2–3 min) and the dual-task assessment (approximately 10 min). Accordingly, the approximate post-exercise timeline was as follows: exercise cessation (0 min), Post saliva collection (0–2 min), tennis accuracy assessments (approximately 2–7 min), single-task 2-back assessment (approximately 7–10 min), and dual-task assessment (approximately 10–20 min). Participants then remained seated until the Post-30 min saliva sample was collected 30 min after exercise cessation. The overall post-exercise assessment sequence was kept constant across participants and was identical in the TSEP and HIIT conditions. Within the tennis accuracy assessment block, the order of forehand, backhand, and serve tests was randomized across participants and then maintained for each participant across all assessment sessions.

**Figure 1:**
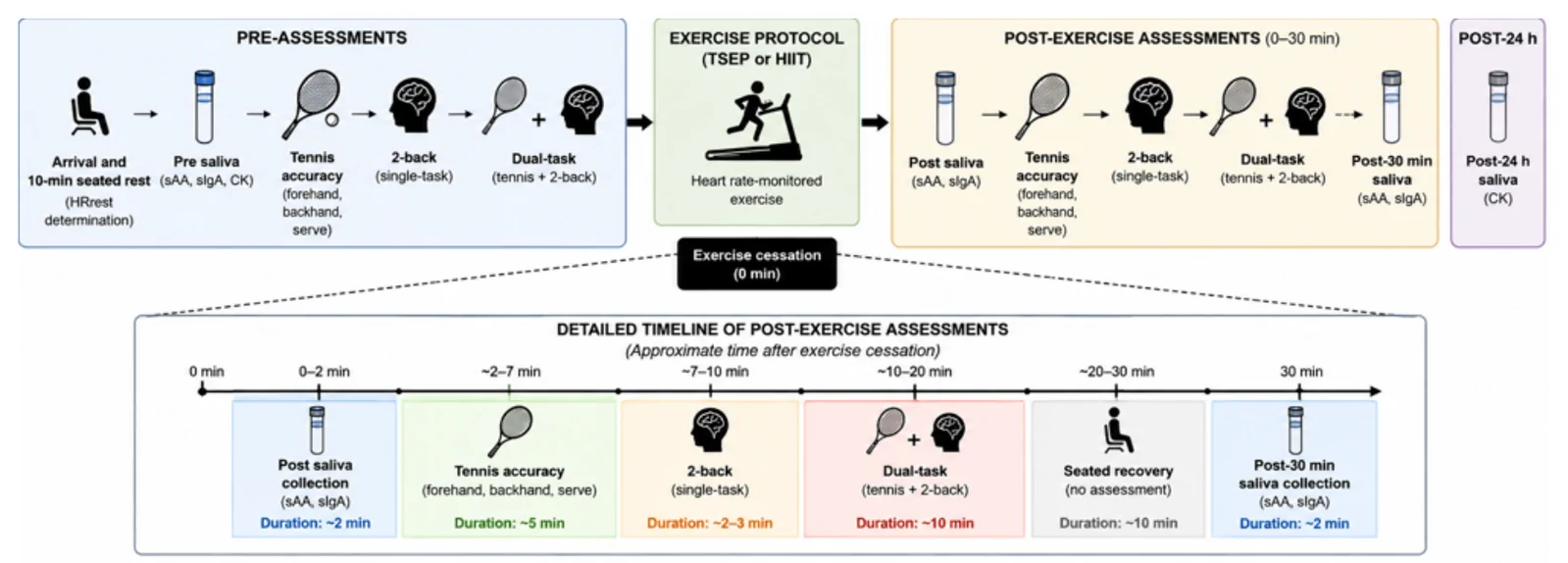
A schematic overview of the experimental protocol and timing of pre- and post-exercise assessments.

All assessments were performed by the same investigator using identical equipment and standardized procedures to maintain methodological consistency. The overall experimental timeline and measurement schedule are illustrated in Figure 1.

### 2.7. Measurements

#### 2.7.1. Anthropometric Parameters

Participants’ height was measured without shoes, standing upright on a flat surface with their heels and both big toes touching, using a stadiometer (SECA, Hamburg, Germany) with an accuracy of 0.01 m. Body weight was measured without shoes and while wearing sports clothing (tracksuit and T-shirt) using an electronic scale (SECA, Germany) with an accuracy of 0.1 kg. Body composition was assessed using a bioelectrical impedance analyzer (InBody 270, InBody Co., Seoul, Republic of Korea), from which body mass index (BMI) was calculated automatically.

#### 2.7.2. Salivary Biomarker Assessment

Salivary sAA, sIgA, and CK were analyzed in this study. Saliva samples for sAA and sIgA analyses were collected at the Pre, Post, and Post-30 min time points, whereas samples for CK analysis were collected at the Pre and Post-24 h time points. Salivary CK was evaluated as an indirect marker of exercise-related muscle stress.

#### 2.7.3. Sample Collection

Participants were instructed to refrain from eating, drinking, brushing their teeth, and chewing gum for at least 30 min before the experimental sessions. Unstimulated whole saliva was collected using the passive drool technique into sterile polypropylene tubes. Approximately 1–2 mL of saliva was obtained from each participant at each collection time point. The Pre saliva sample was collected immediately after the 10 min seated resting period used to determine HRrest. No additional 10 min seated stabilization period was applied before the immediate Post or Post-30 min saliva samples. The immediate Post sample was collected within approximately 0–2 min following exercise cessation. For the Post-24 h CK assessment, participants rinsed their mouths with water for approximately 30 s and then remained seated quietly for 10 min before saliva collection. To minimize the influence of circadian variation on biomarker concentrations, all samples were collected between 09:00 and 11:00 a.m. Samples were visually inspected for evidence of blood contamination or oral bleeding. Samples showing visible blood contamination or oral bleeding were predefined to be excluded from the analyses; however, no samples met these exclusion criteria, and therefore no saliva samples were excluded for this reason.

#### 2.7.4. Sample Processing and Storage

Immediately after collection, saliva samples were transported to the laboratory under refrigerated conditions (4 °C). Samples were centrifuged at 1500× *g* for 15 min to remove cellular debris. The resulting supernatant was transferred into sterile microtubes and stored at −80 °C until the biochemical analyses.

#### 2.7.5. Biomarker Analysis

All biochemical analyses were performed at the Molecular Biology Laboratory of Fırat University. Salivary sIgA and CK concentrations were determined using commercially available enzyme-linked immunosorbent assay (ELISA) kits [13,14], while sAA activity was measured using a kinetic enzymatic assay, according to the manufacturer’s instructions (YL Biont, Shanghai, China). The specific catalog numbers, lower limits of detection (LOD), and assay dynamic ranges were as follows:

sAA: Cat. No: YLA0819HU; LOD: 10.13 U/L; Assay Range: 20–6000 U/L.

sIgA: Cat. No: YLA0762HU; LOD: 0.01 μg/mL; Assay Range: 0.02–7 μg/mL.

CK: Cat. No: YLA1344HU; LOD: 0.57 U/L; Assay Range: 1–300 U/L.

Before analysis, frozen saliva samples were thawed once at room temperature and centrifuged at 1500× *g* for 15 min to remove any remaining cellular debris. The resulting supernatant was collected for biochemical analysis, and repeated freeze–thaw cycles were avoided. Salivary CK samples with values exceeding the upper limit of the assay range were appropriately diluted and reanalyzed according to the manufacturer’s instructions. The corresponding dilution factors were applied when calculating the final CK concentrations.

To minimize inter-assay variability, all samples obtained from a given participant across conditions and time points were analyzed in the same assay batch and, where applicable, on the same microplate. All standards, quality controls, and samples were analyzed in duplicate. For the ELISA analyses, optical density was measured at 450 nm using a microplate reader, and biomarker concentrations were calculated from the respective standard curves according to the manufacturer’s protocols. Salivary alpha-amylase (sAA) activity was determined using the kinetic procedure specified by the manufacturer.

According to the manufacturer’s specifications, the intra-assay coefficients of variation (CVs) were <8% and inter-assay CVs were <10% for all assays. Laboratory personnel were strictly blinded to participant conditions (TSEP vs. HIIT) and time points throughout the analytical procedures. All biomarker concentrations and units were verified against the respective standard curves and manufacturer specifications. No biomarker measurements were missing, and all available biomarker data were included in the statistical analyses.

#### 2.7.6. Tennis Performance Assessment

**Figure 2:**
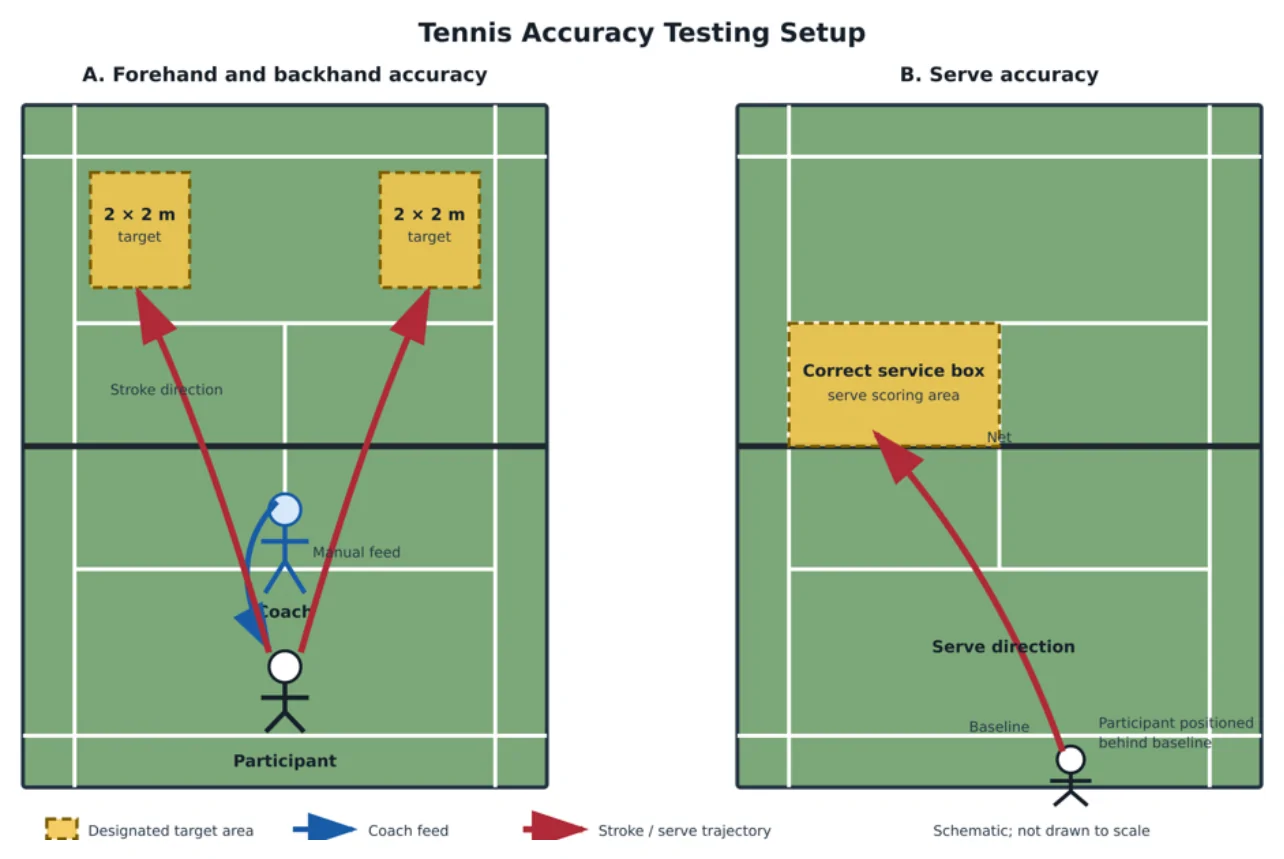
A schematic representation of the tennis accuracy testing setup. During the forehand and backhand assessments, balls were manually delivered by the coach, and participants directed their strokes toward the designated 2 × 2 m target areas. During the serve assessment, participants served from behind the baseline and directed the ball diagonally toward the correct service box. The shaded areas indicate the scoring zones. The diagram is not drawn to scale.

Tennis-specific performance was assessed using a stroke accuracy test adapted from previously described tennis performance protocols [15,16]. Participants performed 10 forehand groundstrokes, 10 backhand groundstrokes, and 10 serves. During the forehand and backhand assessments, participants aimed their strokes toward designated 2 × 2 m target areas located in the opposite court. During the serve assessment, participants served from behind the baseline and directed each serve diagonally toward the correct service box. The locations of the participants, coach, and scoring areas are illustrated in Figure 2.

Before data collection, participants completed 10 practice attempts for each stroke type. A 1 min rest interval was provided between the forehand, backhand, and serve accuracy assessments. The order of the forehand, backhand, and serve tests was randomized across participants and then maintained for each participant across all assessment sessions to minimize potential order effects while ensuring consistency across repeated measurements.

During the forehand and backhand assessments, balls were manually delivered by the same experienced tennis coach from a fixed court position. The coach attempted to maintain consistent ball placement, trajectory, and feeding rhythm across participants and testing sessions. However, ball speed and trajectory were not instrumentally quantified, and a ball machine was not used. Serves were self-initiated by the participants and were not coach-fed.

A forehand or backhand stroke was considered successful when the ball landed within the corresponding 2 × 2 m target area. A serve was considered successful when the ball landed within the correct diagonal service box. Each successful attempt was awarded one point, whereas an unsuccessful attempt received zero points. Performance for each stroke type was expressed as the percentage of successful attempts using the following equation:

Success rate (%) = (Number of successful shots/10) × 100

Separate success percentages were calculated for forehand, backhand, and serve performance. Because each outcome was based on 10 attempts, possible scores ranged from 0% to 100% in increments of 10 percentage points. All stroke outcomes were evaluated by the same assessor using predefined spatial scoring criteria. The assessor was blinded to the exercise condition. Study-specific intra-rater and test–retest reliability were not evaluated. All assessments were conducted on the same tennis court under comparable environmental conditions using the same equipment.

#### 2.7.7. Cognitive and Dual-Task Performance Assessment

Cognitive performance was evaluated using an adapted auditory 2-back task based on the widely used 2-back paradigm, which requires the continuous monitoring and updating of information [17,18]. The outcome is described as “working memory-related” because performance may also involve attention, auditory processing, response selection, and motor responding. Before data collection, all participants completed a familiarization session involving the tennis-specific accuracy assessments, auditory 2-back task, and required response procedure to reduce variability associated with task unfamiliarity [18,19].

#### 2.7.8. Single-Task Cognitive Performance

Working memory-related cognitive performance was assessed using an adapted, verbally administered auditory 2-back task based on the widely used 2-back paradigm [17,18]. Participants stood upright while the same trained investigator verbally presented a sequence of 30 letters containing 15 target stimuli. To minimize potential learning and memorization effects, different letter sequences were used across participants, and each participant received different sequences during the TSEP and HIIT experimental sessions. All sequences were matched for the total number of stimuli (30), number of target stimuli (15), and presentation interval. Participants were instructed to raise one hand whenever the currently presented letter matched the letter presented two positions earlier. Standardized instructions were used, and consecutive letters were presented at approximately 2 s intervals.

A correctly detected target stimulus was classified as a hit, whereas a target stimulus without a response was classified as a miss. Target detection accuracy was calculated as follows:

Target detection accuracy (%) = (Number of correctly detected target letters/15 target letters) × 100

False-alarm responses to non-target stimuli were not systematically recorded; therefore, false alarms and correct rejections could not be incorporated into the scoring, and reaction time was not measured. Accordingly, the outcome represents the proportion of target stimuli correctly detected rather than a comprehensive measure incorporating all possible response types.

#### 2.7.9. Single-Task Tennis Performance

Single-task tennis performance consisted of the tennis-specific performance assessment described above. Participants completed 30 tennis strokes—10 forehand groundstrokes, 10 backhand groundstrokes, and 10 serves—without performing a concurrent cognitive task. A forehand or backhand stroke was classified as successful when the ball landed within the corresponding 2 × 2 m target area, whereas a serve was classified as successful when it landed within the correct diagonal service box. Forehand, backhand, and serve accuracy were calculated separately as the percentage of successful attempts relative to the 10 attempts performed for each stroke type.

#### 2.7.10. Dual-Task Performance

Dual-task performance was assessed by simultaneously evaluating tennis-specific motor performance and working memory-related cognitive performance using an adapted cognitive–motor dual-task paradigm [20,21]. Participants completed 30 tennis strokes—10 forehand groundstrokes, 10 backhand groundstrokes, and 10 serves—while concurrently performing an auditory 2-back task. The same trained investigator verbally presented a fixed sequence of 30 letters containing 15 target stimuli, with one letter presented immediately before each tennis stroke. The same stimulus sequence was used for all participants and across all experimental sessions to maintain consistency in the number, position, and order of target stimuli.

Motor performance under dual-task conditions was assessed independently of cognitive performance. Under dual-task conditions, a forehand or backhand stroke was classified as successful when the ball landed within the corresponding 2 × 2 m target area, whereas a serve was classified as successful when it landed within the correct diagonal service box. Forehand, backhand, and serve accuracy were calculated separately as follows:

Dual-task stroke accuracy (%) = (Number of successful strokes/10 attempts) × 100

Cognitive performance under dual-task conditions was also assessed independently of motor performance. A correctly detected target stimulus was classified as a hit, whereas a target stimulus without a response was classified as a miss. Dual-task target detection accuracy was calculated as follows:

Dual-task target detection accuracy (%) = (Number of correctly identified target letters/15) × 100

False-alarm responses to non-target stimuli were not systematically recorded; therefore, false alarms and correct rejections could not be incorporated into the cognitive score, and reaction time was not measured.

A combined dual-task response was classified as successful only when both components were completed correctly: the participant correctly identified a target letter and the corresponding tennis stroke met the stroke-specific accuracy criterion—that is, the ball landed within the designated 2 × 2 m target area for a forehand or backhand stroke or within the correct diagonal service box for a serve. Combined dual-task accuracy was calculated as follows:

Combined dual-task accuracy (%) = (Number of successful combined responses/15 target stimuli) × 100

Accordingly, the dual-task outcome variables were forehand accuracy (%), backhand accuracy (%), serve accuracy (%), target detection accuracy (%), and combined dual-task accuracy (%). The separated motor and cognitive outcomes were analyzed alongside the combined score to distinguish component-specific changes that could not be identified from the combined outcome alone.

#### 2.7.11. Dual-Task Cost Calculation

Dual-task costs (DTCs) were calculated separately for forehand, backhand, serve, and cognitive accuracy by comparing each participant’s dual-task performance with the corresponding single-task performance at the same condition and time point. Because higher accuracy values indicated better performance, DTC was calculated as follows:

DTC (%) = [(single-task accuracy − dual-task accuracy)/single-task accuracy] × 100

Positive values indicated poorer performance under dual-task conditions, a value of zero indicated no difference between single- and dual-task performance, and negative values indicated that dual-task accuracy exceeded the corresponding single-task accuracy. Motor DTCs were calculated separately for forehand, backhand, and serve accuracy, while cognitive DTC was calculated using single-task and dual-task 2-back target detection accuracy. DTC was treated as missing when the corresponding single-task accuracy was zero because a relative cost could not be calculated. DTC outcomes were calculated at the individual-participant level and are reported descriptively in Supplementary Table S3.

### 2.8. Statistical Analysis

Statistical analyses were performed using IBM SPSS Statistics (version 25; IBM Corp., Armonk, NY, USA). Descriptive statistics are presented as mean ± standard deviation (SD). Potential outliers in the observed continuous outcome data were evaluated using boxplots. For each fitted linear mixed-effects model (LMM), the normality assumption was assessed using the model residuals rather than the raw outcome scores. Standardized residuals were examined separately for each LMM using visual inspection of Q–Q plots and histograms, supported by the Shapiro–Wilk test. Because the accuracy outcomes were analyzed using binomial generalized linear mixed-effects models (GLMMs), normality of the outcome values or residuals was not assumed for these models. All tests were two-sided, and the nominal statistical significance threshold was set at *p* < 0.05.

Given the randomized two-period crossover design, different mixed-effects modeling approaches were used according to the distribution and scale of the outcomes. Continuous salivary biomarker outcomes (sAA, sIgA, and CK) were analyzed using LMMs. Condition (TSEP vs. HIIT), time, period (Period 1 vs. Period 2), sequence (TSEP–HIIT vs. HIIT–TSEP), and the Condition × Time interaction were included as fixed effects, and participant was included as a random intercept to account for repeated observations within participants. For the LMMs, repeated measurements within participants were modeled using a compound symmetry covariance structure, and model parameters were estimated using restricted maximum likelihood (REML). The statistical significance of fixed effects was evaluated using Type III tests.

Bounded accuracy outcomes based on the number of successful responses or trials were analyzed using binomial GLMMs with a logit link. These outcomes included single-task tennis accuracy (forehand, backhand, and serve), single-task 2-back target detection accuracy, dual-task motor accuracy (forehand, backhand, and serve), dual-task 2-back target detection accuracy, and combined dual-task accuracy. Condition, time, period, sequence, and the Condition × Time interaction were included as fixed effects, and participant was included as a random intercept.

Before the main outcome analyses, Pre values were examined separately to evaluate potential systematic baseline period, sequence, and residual carryover effects. For each outcome, an exploratory LMM including period, sequence, and the Period × Sequence interaction as fixed effects and participant as a random intercept was fitted to the Pre values. The main effect of period was used to assess whether Pre values differed systematically between Periods 1 and 2, while the main effect of sequence was used to assess baseline differences between participants assigned to the TSEP–HIIT and HIIT–TSEP sequences. The Period × Sequence interaction was examined as an indicator of whether changes in Pre values between periods differed according to the treatment received in the preceding period, which would be compatible with a potential residual carryover effect. The absence of significant period and Period × Sequence effects was interpreted as indicating no statistical evidence of systematic baseline period differences or residual carryover after the 7-day washout period. Given the small number of participants in each sequence, these analyses were considered exploratory, and nonsignificant effects were not interpreted as definitive evidence of the absence of carryover.

No single outcome was prospectively designated as the primary outcome. Because multiple biomarker, cognitive, and tennis-specific performance outcomes were examined, all outcome analyses were considered exploratory. Bonferroni adjustments were applied to pairwise and simple-effects comparisons within each fitted model; however, no additional multiplicity correction was applied across the separate outcome models. Accordingly, fixed-effect *p*-values are reported as nominal and, particularly when close to the 0.05 threshold, were interpreted cautiously. Model-derived estimates and between-condition contrasts were evaluated together with their 95% confidence intervals, as appropriate to the fitted model. The findings should therefore be considered hypothesis-generating and require confirmation in larger, prospectively powered studies.

The number of time points included in each primary model depended on the outcome. For salivary sAA, and sIgA, time comprised Pre, Post, and Post-30 min measurements. For salivary CK, time comprised Pre and Post-24 h measurements. For single-task tennis performance (forehand, backhand, and serve accuracy), dual-task motor performance (forehand, backhand, and serve accuracy), single-task 2-back target detection accuracy, dual-task 2-back target detection accuracy, and combined dual-task accuracy, time comprised Pre and Post measurements.

To quantify the interference associated with performing the motor and cognitive tasks concurrently, dual-task costs (DTCs) were calculated separately for the motor and cognitive components at the individual-participant level. Motor DTCs were calculated separately for forehand, backhand, and serve accuracy by comparing performance under dual-task conditions with the corresponding single-task performance at the same condition and time point. Cognitive DTC was calculated by comparing dual-task target detection accuracy with the corresponding single-task 2-back target detection accuracy. Because higher accuracy values indicated better performance, DTC was calculated as follows:

DTC (%) = [(Single-task performance − Dual-task performance)/Single-task performance] × 100

Positive DTC values indicated poorer performance under dual-task conditions, a value of zero indicated no difference between single- and dual-task performance, and negative values indicated that dual-task accuracy exceeded the corresponding single-task accuracy. DTC was treated as missing when the corresponding single-task accuracy was zero because a relative cost could not be calculated. This occurred for one backhand observation at HIIT Post.

Relative motor and cognitive DTC outcomes were not subjected to inferential statistical testing. Because several ratio-derived DTC distributions showed substantial departures from normality, the values were sensitive to the corresponding single-task denominators, and one backhand DTC value was undefined because the corresponding single-task accuracy was zero, DTC outcomes were summarized descriptively and treated as exploratory. Descriptive DTC results are presented in Supplementary Table S3.

Estimated marginal means or model-derived estimates, as appropriate to the fitted model, were obtained for interpretation of the mixed-effects analyses. Pairwise comparisons were adjusted using the Bonferroni method. For significant Condition × Time interactions, Bonferroni-adjusted simple-effects comparisons were performed to examine differences between TSEP and HIIT at each time point and changes over time within each condition. Period and sequence effects were retained in all primary models to account for potential systematic effects associated with the crossover design. Given the small number of participants assigned to each intervention sequence, period-, sequence-, and carryover-related findings were interpreted cautiously.

## 3. Results

Actual heart rate responses recorded during the exercise protocols are presented in Supplementary Table S4. Mean HR was 167.5 ± 1.8 beats·min^−1^ during TSEP and 181.7 ± 1.6 beats·min^−1^ during HIIT, corresponding to 78.6 ± 1.0% and 90.3 ± 0.8% of HRR, respectively. Peak HR was 179.3 ± 2.0 beats·min^−1^ during TSEP and 190.6 ± 1.6 beats·min^−1^ during HIIT, corresponding to peak HRR values of 88.3 ± 1.2% and 97.6 ± 0.8%, respectively.

As presented in Table 3, for sAA, the linear mixed-effects model revealed a significant main effect of time, F(2, 54) = 61.288, *p* < 0.001. Bonferroni-adjusted pairwise comparisons indicated that sAA increased significantly from Pre to Post (*p* < 0.001) and remained significantly elevated at Post-30 min compared with Pre (*p* < 0.001). However, sAA decreased significantly from Post to Post-30 min (*p* < 0.001). The main effect of condition, F(1, 54) = 0.833, *p* = 0.383, and the Condition × Time interaction, F(2, 54) = 0.350, *p* = 0.707, were not significant. Neither the period effect, F(1, 54) = 0.004, *p* = 0.949, nor the sequence effect, F(1, 10) = 0.315, *p* = 0.587, was significant.

For sIgA, the linear mixed-effects model revealed a significant main effect of time, F(2, 54) = 62.984, *p* < 0.001. Bonferroni-adjusted pairwise comparisons indicated that sIgA decreased significantly from baseline to Post (*p* < 0.001), subsequently increased significantly from Post to Post-30 min (*p* < 0.001), but remained significantly below baseline at Post-30 min (*p* < 0.001). The main effect of condition, F(1, 54) = 1.681, *p* = 0.200, and the Condition × Time interaction, F(2, 54) = 0.848, *p* = 0.434, were not significant. Neither the period effect, F(1, 54) = 0.029, *p* = 0.866, nor the sequence effect, F(1, 10) = 0.695, *p* = 0.424, was significant.

Because baseline sIgA showed a significant Period × Sequence interaction, a sensitivity analysis was conducted using changes from Pre at Post and Post-30 min. The change-score analysis revealed a significant effect of time, *F*(1, 42) = 36.264, *p* < 0.001. However, the condition effect, *F*(1, 42) = 3.534, *p* = 0.067, and the Condition × Time interaction, *F*(1, 42) = 0.063, *p* = 0.804, were not significant. Neither the period effect, *F*(1, 42) = 2.815, *p* = 0.101, nor the sequence effect, *F*(1, 42) = 1.485, *p* = 0.230, was significant. Thus, the conclusion that the temporal pattern of the sIgA response did not differ significantly between TSEP and HIIT was retained in the change-score sensitivity analysis.

As shown in Table 4, the linear mixed-effects model for salivary CK revealed significant main effects of condition, F(1, 32) = 9.158, *p* = 0.005, and time, F(1, 32) = 352.449, *p* < 0.001, together with a significant Condition × Time interaction, F(1, 32) = 5.009, *p* = 0.032. Neither the period effect, F(1, 32) = 0.031, *p* = 0.860, nor the sequence effect, F(1, 10.001) = 0.071, *p* = 0.796, was significant. Bonferroni-adjusted simple-effects comparisons showed no significant difference between TSEP and HIIT at Pre (estimated marginal means: 158 vs. 169 U·L^−1^, respectively; *p* = 0.581). At Post-24 h, however, CK was significantly higher following HIIT than following TSEP (480 vs. 403 U·L^−1^, respectively; *p* = 0.001). CK increased significantly from Pre to Post-24 h following both TSEP (estimated mean change = 245 U·L^−1^, *p* < 0.001) and HIIT (estimated mean change = 311 U·L^−1^, *p* < 0.001). The magnitude of the Pre-to-Post-24 h increase was significantly greater following HIIT than following TSEP, as indicated by the significant Condition × Time interaction.

Because baseline CK showed a significant Period × Sequence interaction, a sensitivity analysis was conducted using Pre-to-Post-24 h change scores. The change-score analysis confirmed a significant condition effect, F(1, 10) = 12.774, *p* = 0.005, whereas the period effect, F(1, 10) = 0.153, *p* = 0.704, and the sequence effect, F(1, 10) = 2.390, *p* = 0.153, were not significant. Thus, the greater CK increase following HIIT was retained in the change-score analysis.

Tennis-specific accuracy outcomes are presented in Table 5. The binomial generalized linear mixed-effects model revealed a significant main effect of time on forehand accuracy, F(1, 42) = 4.366, *p* = 0.043. The model-estimated probability of a successful forehand decreased from Pre to Post when averaged across conditions. However, neither the main effect of condition, F(1, 42) = 0.827, *p* = 0.368, nor the Condition × Time interaction, F(1, 42) = 1.734, *p* = 0.195, was significant. The period, F(1, 42) = 0.291, *p* = 0.593, and sequence effects, F(1, 42) = 0.031, *p* = 0.861, were also not significant.

For backhand accuracy, a significant main effect of time was observed, F(1, 42) = 6.364, *p* = 0.016, indicating an overall reduction in the model-estimated probability of a successful backhand from Pre to Post. The main effect of condition, F(1, 42) = 1.008, *p* = 0.321, and the Condition × Time interaction, F(1, 42) = 0.438, *p* = 0.512, were not significant. Neither the period effect, F(1, 42) = 0.999, *p* = 0.323, nor the sequence effect, F(1, 42) = 0.070, *p* = 0.793, was significant.

For serve accuracy, no significant main effect of time, F(1, 42) = 3.360, *p* = 0.074, or condition, F(1, 42) = 1.221, *p* = 0.276, was found. The Condition × Time interaction was also not significant, F(1, 42) = 0.310, *p* = 0.581. Similarly, neither the period effect, F(1, 42) = 0.034, *p* = 0.854, nor the sequence effect, F(1, 42) = 0.000, *p* = 0.999, was significant. Thus, although the estimated success probabilities generally declined from Pre to Post, the magnitude of change did not differ significantly between TSEP and HIIT for any of the three stroke outcomes.

The single-task 2-back and dual-task performance outcomes are presented in Table 6. For single-task 2-back target detection accuracy, the binomial generalized linear mixed-effects model revealed a significant main effect of time, F(1, 42) = 23.862, *p* < 0.001, indicating an overall reduction in the estimated probability of correctly detecting a target from Pre to Post. However, neither the main effect of condition, F(1, 42) = 3.541, *p* = 0.067, nor the Condition × Time interaction, F(1, 42) = 1.555, *p* = 0.219, was statistically significant.

For the dual-task motor outcomes, a significant main effect of time was observed only for backhand accuracy, F(1, 42) = 4.800, *p* = 0.034, indicating an overall reduction in the estimated probability of a successful backhand from Pre to Post. The main effects of time were not significant for forehand, F(1, 42) = 2.524, *p* = 0.120, or serve accuracy, F(1, 42) = 2.575, *p* = 0.116. The main effects of condition were not significant for forehand, F(1, 42) = 0.078, *p* = 0.782; backhand, F(1, 42) = 0.441, *p* = 0.510; or serve accuracy, F(1, 42) = 1.085, *p* = 0.303. Similarly, none of the Condition × Time interactions were significant for forehand, F(1, 42) = 0.711, *p* = 0.404; backhand, F(1, 42) = 0.099, *p* = 0.755; or serve accuracy, F(1, 42) = 0.018, *p* = 0.895.

Dual-task cognitive accuracy showed a significant main effect of time, F(1, 42) = 15.700, *p* < 0.001, indicating an overall reduction in the estimated probability of correctly detecting a target from Pre to Post. However, neither the main effect of condition, F(1, 42) = 0.435, *p* = 0.513, nor the Condition × Time interaction, F(1, 42) = 0.636, *p* = 0.430, was statistically significant. Similarly, combined dual-task accuracy showed a significant main effect of time, F(1, 42) = 6.090, *p* = 0.018, but neither the main effect of condition, F(1, 42) = 0.783, *p* = 0.381, nor the Condition × Time interaction, F(1, 42) = 0.030, *p* = 0.862, was significant. No significant period or sequence effects were observed for any of the single-task or dual-task outcomes (all *p* > 0.05). Overall, although several outcomes declined from Pre to Post, the magnitude of these temporal changes did not differ significantly between TSEP and HIIT.

Period and sequence effects for the primary outcome models are reported in Table 3, Table 4, Table 5 and Table 6. No statistically significant sequence effects were detected. Diagnostic analyses of Pre values showed no significant Period × Sequence interaction for most outcomes; however, significant interactions were observed for sIgA, *F*(1,10) = 5.149, *p* = 0.047, and salivary CK, *F*(1,10) = 6.439, *p* = 0.029 (Supplementary Table S1). Sensitivity analyses for these outcomes did not materially alter the primary conclusions. Given the small number of participants within each sequence, the absence of significant sequence effects should not be interpreted as definitive evidence that carryover was absent.

## 4. Discussion

The present study compared the acute effects of a 40 min tennis-specific exercise protocol (TSEP) and a duration-matched 40 min high-intensity interval training (HIIT) protocol on salivary biomarkers and tennis-specific, cognitive, and dual-task performance in amateur male tennis players. The principal findings were that: (i) both protocols produced significant time-dependent changes in salivary alpha-amylase (sAA) and secretory immunoglobulin A (sIgA), without significant condition or Condition × Time interaction effects; (ii) salivary creatine kinase (CK) increased from Pre to Post-24 h following both protocols, with a significantly greater increase following HIIT; and (iii) the binomial generalized linear mixed models identified significant overall time effects for single-task forehand and backhand accuracy and single-task 2-back target detection accuracy, whereas the time effect for single-task serve accuracy was not statistically significant. However, none of the single-task performance outcomes showed a significant Condition × Time interaction, providing insufficient evidence that their changes differed between TSEP and HIIT.

Among the separated dual-task components, significant overall time effects were observed for backhand and cognitive accuracy, whereas the time effects for forehand and serve accuracy were not statistically significant. Combined dual-task accuracy also showed a significant overall time effect. Nevertheless, none of the separated or combined dual-task outcomes showed a significant Condition × Time interaction. Sequential Bonferroni-adjusted comparisons indicated significant Pre-to-Post reductions in single-task forehand and backhand accuracy following TSEP and in single-task 2-back accuracy following both protocols. For the dual-task outcomes, cognitive accuracy decreased significantly following both protocols, whereas the within-condition changes in forehand, backhand, serve, and combined accuracy did not reach statistical significance after adjustment. Although single-task 2-back accuracy was significantly lower following TSEP than HIIT at Post, its nonsignificant Condition × Time interaction means that this simple comparison should not be interpreted as evidence of a differential change between protocols.

Collectively, these findings indicate that both exercise protocols were followed by declines in selected technical and cognitive outcomes, but the current analyses provide insufficient evidence that the magnitude of these changes differed between TSEP and HIIT. Similarly, no statistically significant between-condition differences were detected in the temporal responses of the measured salivary stress and mucosal immune biomarkers. In contrast, the greater Post-24 h salivary CK response following HIIT suggests that the exercise modes may produce different delayed salivary biochemical responses. However, salivary CK should not be interpreted as a direct measure of structural skeletal muscle damage.

The study hypothesized that HIIT would elicit greater physiological stress and greater impairment in tennis-specific performance than TSEP. This hypothesis was not supported overall. The absence of significant Condition × Time interactions for salivary alpha-amylase (sAA) and secretory immunoglobulin A (sIgA) provided no evidence that HIIT elicited greater temporal responses in these selected salivary stress and mucosal immune biomarkers. Although the greater Post-24 h increase in salivary CK following HIIT provided outcome-specific support for a greater delayed salivary CK response, this finding should not be generalized as evidence of greater overall physiological stress or structural skeletal muscle damage. The performance-related hypothesis was also not supported. Although adjusted within-condition comparisons identified reductions in selected technical and cognitive outcomes, none of the single-task, separated dual-task, or combined dual-task outcomes showed a significant Condition × Time interaction. Therefore, the present analyses provide insufficient evidence that the magnitude of performance change differed between TSEP and HIIT. Given the small sample size, these nonsignificant interactions should be interpreted as an absence of statistically reliable evidence of a between-protocol difference rather than evidence of equivalent effects.

One important finding was that no significant differences were detected between TSEP and HIIT in the temporal responses of salivary alpha-amylase (sAA) or secretory immunoglobulin A (sIgA). sAA increased significantly from Pre to Post and subsequently decreased significantly at Post-30 min, while remaining significantly elevated relative to Pre. In contrast, sIgA decreased significantly from Pre to Post and subsequently increased significantly at Post-30 min, although it remained significantly below Pre. These findings indicate that both biomarkers showed significant time-dependent responses, but their temporal patterns did not differ significantly between TSEP and HIIT. Thus, the present findings provide no evidence that the temporal responses of these selected salivary stress and mucosal immune biomarkers differed between TSEP and HIIT. However, the absence of significant between-condition differences should not be interpreted as evidence of physiological equivalence, particularly because comprehensive measures of internal and external exercise load were not obtained.

The increase in sAA activity is consistent with increased sympathetic nervous system activity during vigorous exercise and with previous reports of rapid elevations following intermittent high-intensity exercise [22,23,24]. The acute reduction in salivary sIgA concentration, followed by partial recovery at Post-30 min, is also consistent with previous findings suggesting that strenuous exercise may temporarily alter mucosal immune responses [23,24,25]. However, salivary flow rate was not measured in the present study. Consequently, the observed changes in sAA activity and sIgA concentration may partly reflect exercise-induced changes in salivary flow and dilution. These findings should therefore be interpreted as changes in salivary sAA activity and sIgA concentration rather than definitive changes in their secretion rates.

Despite the sport-specific characteristics of TSEP, no evidence was found that it elicited different sAA or sIgA responses from duration-matched HIIT. Although TSEP involved repeated accelerations, decelerations, multidirectional movements, and technical actions, these characteristics did not produce significantly different temporal responses in the selected salivary biomarkers. Nevertheless, the absence of significant condition and Condition × Time effects should not be interpreted as evidence of physiological equivalence between the protocols. The prescribed intensity distributions differed between TSEP and HIIT, and comprehensive quantitative measures of internal and external exercise load were not obtained. Consequently, the protocols may have differed in physiological or mechanical demands that were not captured by the selected salivary biomarkers.

In contrast to sAA and sIgA, salivary CK exhibited a condition-dependent temporal response. Salivary CK increased significantly from Pre to Post-24 h following both protocols; however, the magnitude of this increase was significantly greater following HIIT than following TSEP. Although salivary CK did not differ between conditions at Pre, it was significantly higher following HIIT at Post-24 h, consistent with the significant Condition × Time interaction. These findings indicate that HIIT elicited a greater delayed salivary CK response than TSEP. However, salivary CK is not an established direct measure of structural skeletal muscle damage, and its physiological interpretation remains less definitive than that of circulating CK. Moreover, complementary measures such as serum CK, myoglobin, muscle soreness, force loss, or muscle imaging were not obtained. Therefore, the greater salivary CK response following HIIT should be interpreted as a potential indicator of a differential exercise-related biochemical response rather than direct evidence of greater skeletal muscle damage.

Several unmeasured mechanisms could plausibly have contributed to the greater salivary CK response following HIIT. Compared with TSEP, HIIT was performed at a higher prescribed intensity distribution and involved repeated bouts of high-intensity running, which may have imposed greater contractile and mechanical demands on the lower-limb musculature [26,27]. Although TSEP also incorporated accelerations, decelerations, changes in direction, and explosive tennis-specific actions, its movement demands were distributed across a wider range of technical actions involving both the upper and lower body. Thus, the protocols may have produced different patterns of muscular loading, potentially contributing to the greater Post-24 h salivary CK response following HIIT. Nevertheless, external mechanical load and neuromuscular strain were not directly quantified, salivary CK may be affected by several physiological and methodological factors, and no complementary measures of skeletal muscle damage were obtained. These proposed mechanisms should therefore be regarded as plausible but speculative.

The greater Post-24 h salivary CK response following HIIT was not accompanied by greater impairment in the measured technical or cognitive performance outcomes. The binomial generalized linear mixed models identified significant overall time effects for single-task forehand and backhand accuracy and single-task 2-back target detection accuracy, whereas the time effect for serve accuracy was not statistically significant. Sequential Bonferroni-adjusted comparisons indicated significant Pre-to-Post reductions in forehand and backhand accuracy following TSEP and in 2-back target detection accuracy following both protocols. However, none of these outcomes showed a significant Condition × Time interaction. Accordingly, the significant within-condition changes should not be interpreted as evidence that one protocol produced greater deterioration than the other. Although single-task 2-back accuracy was significantly lower following TSEP than HIIT at Post, the corresponding interaction was not significant, and this simple comparison should therefore be interpreted cautiously.

These findings indicate that the magnitude of impairment in tennis-specific or cognitive performance cannot be inferred from the salivary CK response alone. Successful tennis performance requires the integration of visual information, movement preparation, footwork, trunk rotation, racket control, and precise movement timing [28]. Repeated performance of these coordinated actions during TSEP may plausibly have contributed to the within-condition reductions observed in forehand and backhand accuracy. Nevertheless, perceptual–motor, coordinative, and neuromuscular demands were not directly assessed, and the statistical analyses did not provide reliable evidence that the magnitude of change differed between TSEP and HIIT. Furthermore, TSEP did not reproduce the full perceptual, anticipatory, tactical, psychological, or emotional demands of competitive match play. The observed changes should therefore be interpreted in relation to the specific technical, coordinative, and movement demands incorporated into the protocol rather than attributed directly to competitive match-play demands.

Previous investigations have shown that tennis-induced fatigue can adversely affect stroke velocity, stroke precision, movement efficiency, and lower-limb neuromuscular function [29,30]. The present results are broadly consistent with evidence that acute exercise may be followed by deterioration in selected tennis-specific performance outcomes. However, the present findings do not establish that TSEP caused greater overall performance impairment than duration-matched running-based HIIT, because none of the technical performance outcomes showed a significant Condition × Time interaction. These performance changes occurred despite the absence of significant condition-dependent temporal responses in sAA and sIgA and despite the greater delayed salivary CK response following HIIT. This pattern reinforces the view that salivary biomarker responses and functional performance outcomes may reflect different aspects of the acute exercise response. Nevertheless, because the protocols differed in prescribed intensity distribution and comprehensive measures of internal and external exercise load were unavailable, the findings should not be interpreted as evidence that the conditions imposed equivalent physiological or mechanical demands.

Although serve accuracy decreased numerically following both protocols, neither the overall time effect nor the Condition × Time interaction reached statistical significance in the binomial generalized linear mixed model. Sequential Bonferroni-adjusted within-condition comparisons were also nonsignificant. The serve is one of the most technically demanding strokes in tennis because its successful execution requires precise coordination of the kinetic chain, extending from the lower limbs through the trunk to the upper extremity [31]. Although neuromuscular coordination, lower-limb force transfer, trunk stabilization, racket control, and movement timing could plausibly influence post-exercise serve performance, these mechanisms were not directly assessed. Therefore, the observed numerical reductions in serve accuracy should be interpreted cautiously and should not be presented as evidence of a differential effect of TSEP and HIIT.

The cognitive findings indicate that acute exercise was followed by reduced target detection accuracy, but they do not establish that the magnitude of this reduction differed between protocols. Single-task 2-back accuracy showed a significant overall time effect and decreased significantly following both TSEP and HIIT in the sequential Bonferroni-adjusted comparisons. Although accuracy was significantly lower following TSEP than HIIT at Post, the Condition × Time interaction was not significant. Consequently, this Post comparison should not be interpreted as demonstrating a greater exercise-induced decline following TSEP. The 2-back paradigm requires continuous monitoring and updating of information and engages working memory-related processes, although performance may also depend on attention, auditory processing, response selection, and motor responding [32]. The observed reductions should therefore be interpreted as changes in working memory-related target detection rather than as an isolated impairment in working memory. Furthermore, because TSEP did not reproduce the full visual, anticipatory, tactical, psychological, or emotional demands of competitive tennis, these findings do not provide direct evidence regarding visual tracking, anticipation, or tactical decision-making during match play.

When dual-task performance was separated into motor and cognitive components, significant overall time effects were observed for backhand and cognitive accuracy, whereas the time effects for forehand and serve accuracy were not statistically significant. Cognitive accuracy decreased significantly following both protocols in the sequential Bonferroni-adjusted comparisons. In contrast, the adjusted within-condition comparisons for dual-task forehand, backhand, and serve accuracy were not significant. Combined dual-task accuracy showed a significant overall time effect, although its adjusted within-condition comparisons did not reach statistical significance. Importantly, none of the separated or combined dual-task outcomes showed a significant Condition × Time interaction, providing insufficient evidence that their changes differed between TSEP and HIIT.

Relative motor and cognitive dual-task costs were presented descriptively in Supplementary Table S3. The direction and magnitude of these ratio-derived values varied across conditions and time points, including a negative mean cognitive DTC following TSEP at Post. However, relative DTC is sensitive to the corresponding single-task value used as its denominator, and single-task cognitive accuracy declined markedly following TSEP. The negative value should therefore not be interpreted as evidence of improved cognitive capacity under dual-task conditions. Because some DTC distributions showed substantial departures from normality, denominator instability was evident, and one backhand DTC value was undefined owing to a zero single-task denominator, no inferential conclusions were drawn from the DTC outcomes. These descriptive findings should be considered exploratory. Previous evidence that prolonged strenuous exercise may impair executive function provides a possible framework for interpreting the observed cognitive reductions, although the underlying mechanisms were not directly assessed in the present study [33].

Given the small sample size, the study may have had limited statistical power to detect and estimate Condition × Time interactions reliably. Accordingly, nonsignificant interactions indicate an absence of statistically reliable evidence of a between-protocol difference rather than evidence of comparable or equivalent effects.

Taken together, the present findings indicate that the measured salivary biomarker responses and technical and cognitive performance outcomes did not change uniformly following the two exercise protocols. The assessment of physiological biomarkers alone may therefore not fully capture the functional consequences of fatigue following tennis-specific exercise. A multidimensional approach combining physiological, biochemical, technical, and cognitive measures may provide a more comprehensive evaluation of acute fatigue and recovery in tennis players.

Because the sample consisted exclusively of young adult male amateur tennis players, these findings should not be generalized directly to female athletes, junior players, older adults, or elite competitors, whose responses may differ according to sex, age, maturation, training status, and competitive level.

## 5. Limitations of the Study

Several limitations should be acknowledged. First, the small sample of 12 young adult amateur male tennis players limited statistical power, precision, and generalizability, particularly for interaction, sequence, and potential carryover effects. Accordingly, nonsignificant Condition × Time and sequence effects should be interpreted as an absence of statistically reliable evidence rather than evidence that the protocols produced equivalent responses or that carryover was definitively absent. Because the sample consisted exclusively of young adult amateur male players, the findings cannot be directly generalized to female players, adolescents, older adults, or elite tennis players. Furthermore, the findings represent acute responses and cannot be generalized to chronic training adaptations.

Second, salivary flow rate and hydration status were not objectively assessed. Although pre-sampling fluid intake was standardized, individual differences in hydration status may have influenced salivary biomarker concentrations. Because salivary flow rate was not measured, changes in sAA activity and sIgA concentration may partly reflect changes in salivary flow or dilution rather than biomarker-specific responses alone. Salivary CK was not supported by serum CK, myoglobin, muscle soreness, force loss, or imaging measures and should therefore not be interpreted as direct evidence of structural skeletal muscle damage.

Third, tennis accuracy was based on only 10 attempts per stroke, producing scores in 10-percentage-point increments and potentially limiting measurement sensitivity and reliability. Although the accuracy outcomes were analyzed as binomial counts using generalized linear mixed models, the small number of attempts restricted the range and precision of the observed scores. Forehand and backhand balls were manually delivered, and ball speed, trajectory, and placement were not objectively quantified. Although predefined scoring criteria and a blinded assessor were used, study-specific intra-rater and test–retest reliability were not evaluated.

Fourth, the adapted auditory 2-back task was verbally administered using a hand-raising response, which may have introduced timing variability and an additional motor component. The fixed letter sequence used during the dual-task assessments may also have produced learning or familiarization effects. False alarms, correct rejections, and reaction times were not recorded, and the reliability of the adapted task was not independently established. The cognitive findings should therefore be interpreted as working memory-related target detection rather than working memory in isolation.

Fifth, although dual-task motor and cognitive components were reported separately, the combined success score could not distinguish cognitive errors from inaccurate strokes and did not fully characterize cognitive–motor trade-offs or task-prioritization strategies. Relative DTC values were sensitive to the corresponding single-task values used as denominators and may have been unstable when single-task performance was low. One backhand DTC value was undefined because the corresponding single-task accuracy was zero, and some ratio-derived DTC distributions showed substantial departures from normality. Consequently, DTC outcomes were presented descriptively without inferential testing and should be regarded as exploratory. In particular, negative DTC values should not be interpreted as evidence of improved performance under dual-task conditions.

Finally, the protocols were matched for duration but differed in prescribed intensity distribution, and comprehensive measures of internal and external exercise load were unavailable. Maximum heart rate was estimated using an age-predicted equation rather than directly measured using a maximal exercise test, which may have introduced individual error into the calculated HRR-based intensity targets. Furthermore, although heart rate was monitored continuously during exercise using a Polar H10 sensor, the complete time-stamped HR time series was not retained for retrospective analysis. Therefore, time spent within predefined HRR zones could not be calculated. Only session-level mean and peak HR values were retained and are reported in Supplementary Table S4. Because heart rate also responds with a physiological delay to rapid changes in exercise intensity, particularly during short high-intensity intervals, these summary measures cannot verify that participants remained within the prescribed HRR target during each individual exercise segment.

## 6. Conclusions

The present study showed that 40 min TSEP and duration-matched HIIT produced significant time-dependent changes in sAA and sIgA, with no significant between-condition differences in their temporal responses. Salivary CK increased from Pre to Post-24 h following both protocols, but the increase was significantly greater following HIIT. This finding should be interpreted as a greater delayed salivary CK response rather than direct evidence of greater structural skeletal muscle damage.

The binomial generalized linear mixed models identified significant overall time effects for single-task forehand and backhand accuracy and single-task 2-back target detection accuracy, whereas the time effect for single-task serve accuracy was not statistically significant. Among the separated dual-task components, significant overall time effects were observed for backhand and cognitive accuracy, and combined dual-task accuracy also showed a significant overall time effect. However, none of the single-task, separated dual-task, or combined dual-task outcomes showed a significant Condition × Time interaction. Therefore, the present analyses provide insufficient evidence that the magnitude of change in technical, cognitive, or dual-task performance differed between TSEP and HIIT. Relative motor and cognitive DTC outcomes were presented descriptively because of non-normality, denominator instability, and one undefined value and should therefore be regarded as exploratory.

Collectively, the findings indicate that the measured salivary biomarker responses and functional performance outcomes did not change uniformly following the two exercise protocols. In particular, the greater delayed salivary CK response following HIIT was not accompanied by statistically reliable evidence of greater technical or cognitive performance impairment. Salivary biomarkers alone may therefore not fully reflect post-exercise changes in tennis-specific technical and working memory-related cognitive performance. However, given the small and homogeneous sample, limited statistical power, methodological limitations of the adapted cognitive and dual-task assessments, ratio dependence of the DTC calculation, limited characterization of internal and external exercise load, and structured nature of TSEP, these findings should be interpreted cautiously. Further studies using larger and more diverse samples, direct mechanistic measurements, and more ecologically valid competitive settings are required to confirm and explain these findings.

## Figures and Tables

**Table 1:** Participant characteristics.

Variables	N	Mean ± SD	Minimum	Maximum
Age (y)	12	22.08 ± 1.56	19	25
Height (cm)	12	174.16 ± 4.62	168	183
Body mass (kg)	12	72.12 ± 9.16	63.5	84.2
Body mass index (kg/m^2^)	12	23.72 ± 2.34	20.37	27.76

**Table 2:** The exercise protocols used in the crossover design.

Phase	TSEP	HIIT	Duration
Warm-up	5 min low-intensity running (30% HRR) + 5 min tennis-specific dynamic stretching + 3 min short rallies and serve-return drills (70% HRR)	5 min low-intensity running (30% HRR) + 5 min dynamic stretching + 3 min light running (50% HRR)	13 min
Main set 1	3 min forehand and backhand groundstrokes with change-of-direction movements and topspin drills (80% HRR) + 2 min net play incorporating slice and topspin combinations (90% HRR) + 1 min high-intensity serve practice with rapid-reaction drills (95% HRR)	Continuous running around the perimeter of the indoor tennis court: 3 min at 90% HRR + 2 min at 95% HRR + 1 min at 100% HRR	6 min
Passive recovery	Seated passive recovery	Seated passive recovery	1 min
Active recovery	Mini-rally play (40% HRR)	Low-intensity jogging (30% HRR)	3 min
Main set 2	Same 6 min sequence as Main set 1	Same 6 min sequence as Main set 1	6 min
Passive recovery	Seated passive recovery	Seated passive recovery	1 min
Cool-down/recovery	5 min low-intensity jogging (30% HRR) + 5 min static stretching	5 min passive recovery + 5 min low-intensity jogging (30% HRR)	10 min
Total	40 min	40 min	40 min

Note. TSEP = tennis-specific exercise protocol; HIIT = high-intensity interval training; HRR = heart rate reserve. Exercise intensity was prescribed using the Karvonen HRR method. Both protocols included two identical 6 min main exercise blocks. The TSEP drills were prescribed according to exercise duration and individualized HRR targets rather than a fixed number of stroke repetitions or predetermined movement distances. Similarly, HIIT running was performed continuously around the perimeter of the indoor tennis court and was prescribed according to exercise duration and individualized HRR targets rather than a fixed running distance.

**Table 3:** Acute salivary stress and immune biomarker responses to TSEP and HIIT in the randomized crossover design.

Variable	TSEP Pre	TSEP Post	TSEP Post- 30 min	HIIT Pre	HIIT Post	HIIT Post- 30 min	Time	Condition	Condition × Time	Period	Sequence
Salivary α-Amylase (U·L^−1^)	427± 149 ^a^	633 ± 230 ^b^	540± 184 ^c^	376 ± 172 ^a^	600± 196 ^b^	522± 174 ^c^	**F(2, 54) = 61.288, *p* < 0.001**	F(1, 54) = 0.833, *p* = 0.383	F(2, 54) = 0.350, *p* = 0.707	F(1, 54) = 0.004, *p* = 0.949	F(1, 10) = 0.315, *p* = 0.587
Secretory IgA (µg·mL^−1^)	5.871± 0.800 ^a^	4.594± 0.591 ^b^	5.469± 0.741 ^c^	6.198± 0.531 ^a^	4.598± 0.596 ^b^	5.549 ± 0.875 ^c^	**F(2, 54) = 62.984, *p* < 0.001**	F(1, 54) = 1.681, *p* = 0.200	F(2, 54) = 0.848, *p* = 0.434	F(1, 54) = 0.029, *p* = 0.866	F(1, 10) = 0.695, *p* = 0.424

Note. The values are presented as mean ± SD. Fixed effects were evaluated using linear mixed-effects models including condition (TSEP vs. HIIT), time (Pre, Post, and Post-30 min), period (Period 1 vs. Period 2), sequence (TSEP–HIIT vs. HIIT–TSEP), and the Condition × Time interaction. Participant was included as a random intercept to account for the clustering of repeated observations within participants. The model parameters were estimated using restricted maximum likelihood. Bonferroni-adjusted pairwise comparisons were conducted following significant main effects of time. Within each biomarker, means with different superscript letters indicate significant differences between time points based on comparisons of the main effect of time, averaged across conditions (*p* < 0.05). TSEP, tennis-specific exercise protocol; HIIT, high-intensity interval training; sAA, salivary alpha-amylase; sIgA, secretory immunoglobulin A; SD, standard deviation. The bold values indicate statistically significant effects (*p* < 0.05).

**Table 4:** Salivary CK responses and linear mixed-effects model results following TSEP and HIIT.

Variable	TSEP Pre	TSEP Post-24 h	HIIT Pre	HIIT Post-24 h	Condition	Time	Condition × Time	Period	Sequence
CK (U·L^−1^)	158 ± 30	403 ± 74	169 ± 39	480 ± 74	**F(1, 32) = 9.158, *p* = 0.005**	**F(1, 32) = 352.449, *p* < 0.001**	**F(1, 32) = 5.009, *p* = 0.032**	F(1, 32) = 0.031, *p* = 0.860	F(1, 10) = 0.071 *p*= 0.796

Note. The values are presented as mean ± standard deviation (SD). Fixed effects were evaluated using a linear mixed-effects model including condition (TSEP vs. HIIT), time (Pre vs. Post-24 h), period (Period 1 vs. Period 2), sequence (TSEP–HIIT vs. HIIT–TSEP), and the Condition × Time interaction. Participant was included as a random intercept to account for the clustering of repeated observations within participants. The model parameters were estimated using restricted maximum likelihood. Bonferroni-adjusted simple-effects comparisons were conducted to examine differences between conditions at each time point and changes over time within each condition. TSEP, tennis-specific exercise protocol; HIIT, high-intensity interval training; CK, creatine kinase; SD, standard deviation; REML, restricted maximum likelihood. The bold values indicate statistically significant effects (*p* < 0.05).

**Table 5:** Binomial generalized linear mixed-effects model results for tennis-specific accuracy following TSEP and HIIT.

Variable	TSEP Pre	TSEP Post	HIIT Pre	HIIT Post	Condition	Time	Condition × Time	Period	Sequence
Forehand accuracy (%)	64.4 (50.6–76.1)	48.3 (34.9–62.0)	62.5 (48.7–74.6)	59.0 (45.1–71.6)	F(1, 42) = 0.827, *p* = 0.368	**F(1, 42) = 4.366, *p* = 0.043**	F(1, 42) = 1.734, *p* = 0.195	F(1, 42) = 0.291, *p* = 0.593	F(1, 42) = 0.031, *p* = 0.861
Backhand accuracy (%)	53.4 (40.9–65.5)	38.6 (27.4–51.1)	55.0 (42.5–67.0)	46.3 (34.2–58.8)	F(1, 42) = 1.008, *p* = 0.321	**F(1, 42) = 6.364, *p* = 0.016**	F(1, 42) = 0.438, *p* = 0.512	F(1, 42) = 0.999, *p* = 0.323	F(1, 42) = 0.070, *p* = 0.793
Serve accuracy (%)	51.7 (41.8–61.4)	40.8 (31.5–50.8)	54.2 (44.3–63.8)	48.3 (38.6–58.2)	F(1, 42) = 1.221, *p* = 0.276	F(1, 42) = 3.360, *p* = 0.074	F(1, 42) = 0.310, *p* = 0.581	F(1, 42) = 0.034, *p* = 0.854	F(1, 42) = 0.000, *p* = 0.999

Note. The values are model-estimated success probabilities expressed as percentages, with 95% confidence intervals in parentheses. Each outcome represented the number of successful strokes among 10 attempts and was analyzed using a binomial generalized linear mixed-effects model with a logit link. Condition (TSEP vs. HIIT), time (Pre vs. Post), period (Period 1 vs. Period 2), sequence (TSEP–HIIT vs. HIIT–TSEP), and the Condition × Time interaction were included as fixed effects. Participant was included as a random intercept. TSEP, tennis-specific exercise protocol; HIIT, high-intensity interval training; CI, confidence interval. The bold values indicate statistically significant effects (*p* < 0.05).

**Table 6:** Binomial generalized linear mixed-effects model results for single-task 2-back target detection and dual-task motor and cognitive performance following TSEP and HIIT.

Variable	TSEP Pre	TSEP Post	HIIT Pre	HIIT Post	Condition	Time	Condition × Time	Period	Sequence
Single-task 2-back target detection accuracy (%)	62.4 (53.1–71.0)	39.2 (30.5–48.6)	64.7 (55.4–73.0)	51.1 (41.7–60.4)	F(1, 42) = 3.541, *p* = 0.067	**F(1, 42) = 23.862, *p* < 0.001**	F(1, 42) = 1.555, *p* = 0.219	F(1, 42) = 0.006, *p* = 0.939	F(1, 42) = 0.229, *p* = 0.635
Dual-task forehand accuracy (%)	52.5 (40.3–64.4)	41.2 (29.9–53.5)	49.9 (37.8–62.0)	46.4 (34.6–58.6)	F(1, 42) = 0.078, *p* = 0.782	F(1, 42) = 2.524, *p* = 0.120	F(1, 42) = 0.711, *p* = 0.404	F(1, 42) = 0.072, *p* = 0.790	F(1, 42) = 0.409, *p* = 0.526
Dual-task backhand accuracy (%)	42.1 (31.1–54.0)	31.1 (21.5–42.5)	43.8 (32.6–55.6)	35.2 (25.0–46.9)	F(1, 42) = 0.441, *p* = 0.510	**F(1, 42) = 4.800, *p* = 0.034**	F(1, 42) = 0.099, *p* = 0.755	F(1, 42) = 0.202, *p* = 0.655	F(1, 42) = 0.426, *p* = 0.517
Dual-task serve accuracy (%)	39.2 (30.5–48.6)	31.6 (23.6–40.9)	43.3 (34.3–52.8)	36.6 (28.2–46.1)	F(1, 42) = 1.085, *p* = 0.303	F(1, 42) = 2.575, *p* = 0.116	F(1, 42) = 0.018, *p* = 0.895	F(1, 42) = 0.006, *p* = 0.938	F(1, 42) = 0.379, *p* = 0.541
Dual-task cognitive accuracy (%)	54.6 (43.6–65.1)	42.4 (32.1–53.4)	55.1 (44.1–65.6)	37.0 (27.3–47.9)	F(1, 42) = 0.435, *p* = 0.513	**F(1, 42) = 15.700, *p* < 0.001**	F(1, 42) = 0.636, *p* = 0.430	F(1, 42) = 1.591, *p* = 0.214	F(1, 42) = 0.411, *p* = 0.525
Combined dual-task accuracy (%)	38.3 (31.3–45.9)	28.9 (22.6–36.1)	34.4 (27.6–41.9)	26.6 (20.5–33.7)	F(1, 42) = 0.783, *p* = 0.381	**F(1, 42) = 6.090, *p* = 0.018**	F(1, 42) = 0.030, *p* = 0.862	F(1, 42) = 0.345, *p* = 0.560	F(1, 42) = 1.114, *p* = 0.297

Note. The values are model-estimated success probabilities expressed as percentages, with 95% confidence intervals in parentheses. The forehand, backhand, and serve outcomes represented the number of successful strokes among 10 attempts. The single-task and dual-task cognitive outcomes and combined dual-task accuracy were calculated relative to 15 target stimuli. The outcomes were analyzed using binomial generalized linear mixed-effects models with a logit link. Condition (TSEP vs. HIIT), time (Pre vs. Post), period (Period 1 vs. Period 2), sequence (TSEP–HIIT vs. HIIT–TSEP), and the Condition × Time interaction were included as fixed effects. Participant was included as a random intercept. TSEP, tennis-specific exercise protocol; HIIT, high-intensity interval training; CI, confidence interval. The bold values indicate statistically significant effects (*p* < 0.05).

## Data Availability

The data presented in this study are available on request from the corresponding author. The data are not publicly available because they contain individual-level data from human participants and their public disclosure could compromise participant privacy and confidentiality.

## Supplementary Materials

The following supporting information can be downloaded at https://www.mdpi.com/article/10.3390/life16091565/s1, Supplementary Table S1. Baseline period, sequence, and Period × Sequence effects for study outcomes. Supplementary Table S2. Estimated within-condition changes and between-condition differences with 95% confidence intervals. Supplementary Table S3. Descriptive changes in relative motor and cognitive dual-task costs following TSEP and HIIT. Supplementary Table S4. Actual heart-rate responses during the TSEP and HIIT protocols.

## Abbreviations

The following abbreviations are used in this manuscript:

TSEP	Tennis-Specific Exercise Protocol
HIIT	High-Intensity Interval Training
HRR	Heart Rate Reserve
CK	Creatine Kinase
sAA	Salivary Alpha-Amylase
sIgA	Secretory Immunoglobulin A
ELISA	Enzyme-Linked Immunosorbent Assay
LMM	Linear Mixed-Effects Model
DTC	Dual-Task Cost
