# Supplementary material for: Acute Salivary Biomarker, Cognitive, and Tennis Performance Responses to Tennis-Specific Exercise Versus HIIT: A Randomized Crossover Study
Source: Life (Basel). 2026 Sep 18;16(9):1565. doi: 10.3390/life16091565 (PMC13608874; doi:10.3390/life16091565)
Supplement: Supplementary file 1 [file life-16-01565-s001.zip › life-4477410-supplementary.pdf]

Supplementary Materials

Supplementary Table S1. Baseline period, sequence, and Period × Sequence effects for study outcomes.

Outcome	Period F (*p*)	Sequence F (*p*)	Period × Sequence F (*p*)
**Salivary α-amylase**	0.009 (0.926)	1.685 (0.223)	1.244 (0.291)
**sIgA**	1.105 (0.318)	1.241 (0.291)	**5.149 (0.047)**
**CK**	0.047 (0.832)	1.851 (0.204)	**6.439 (0.029)**
**Single-task forehand accuracy**	0.000 (0.995)	0.006 (0.937)	0.075 (0.787)
**Single-task backhand accuracy**	0.000 (0.996)	0.000 (0.994)	0.069 (0.796)
**Single-task serve accuracy**	0.017 (0.898)	0.016 (0.899)	0.151 (0.702)
**Single-task 2-back target detection accuracy**	0.042 (0.839)	0.526 (0.477)	0.189 (0.668)
**Dual-task forehand accuracy**	0.018 (0.896)	0.072 (0.792)	0.154 (0.699)
**Dual-task backhand accuracy**	0.000 (0.998)	0.046 (0.833)	0.069 (0.796)
**Dual-task serve accuracy**	0.018 (0.895)	0.018 (0.895)	0.430 (0.519)
**Dual-task cognitive accuracy**	0.104 (0.750)	0.166 (0.688)	0.011 (0.919)
**Combined dual-task accuracy**	0.117 (0.736)	0.308 (0.585)	0.597 (0.449)

Note. Pre values were analyzed separately to examine baseline period, sequence, and Period × Sequence effects. Biomarker outcomes were analyzed using linear mixed-effects models, whereas accuracy outcomes were analyzed using binomial generalized linear mixed-effects models with a logit link. Period, sequence, and the Period × Sequence interaction were included as fixed effects, with participant included as a random intercept. All tests had 1 numerator degree of freedom. The denominator degrees of freedom were 10 for the biomarker models and 20 for the binomial accuracy models. The Period × Sequence interaction was examined to identify sequence-dependent changes in Pre values between periods that might be compatible with a potential residual carryover effect. Given the small number of participants in each sequence, these analyses were considered exploratory and interpreted cautiously. Bold values indicate statistically significant effects ($p < 0.05$). sIgA, secretory immunoglobulin A; CK, creatine kinase.

Baseline period, sequence, and potential residual carryover effects are presented in Supplementary Table S1. No significant main effects of period or sequence were detected for any outcome (all $p > 0.05$). The Period × Sequence interactions were not significant for salivary α-amylase or any of the single-task or dual-task accuracy outcomes (all $p > 0.05$). However, significant Period × Sequence interactions were observed for baseline sIgA, $F(1, 10) = 5.149$, $p = 0.047$, and salivary CK, $F(1, 10) = 6.439$, $p = 0.029$, indicating that sequence-dependent baseline effects could not be excluded for these biomarkers. Baseline sIgA increased slightly from Period 1 to Period 2 in the TSEP–HIIT sequence (5.746 to 5.922 $\mu g \cdot mL^{-1}$) but decreased in the HIIT–TSEP sequence (6.475 to 5.996 $\mu g \cdot mL^{-1}$). Similarly, baseline CK increased from 144 to 157 $U \cdot L^{-1}$ in the TSEP–HIIT sequence but decreased from 182 to 171 $U \cdot L^{-1}$ in the HIIT–TSEP sequence.

Because these baseline interactions were significant, change-score sensitivity analyses were conducted. For sIgA, the analysis of changes from Pre at Post and Post-30 min revealed a significant effect of time, $F(1, 42) = 36.264$, $p < 0.001$. However, neither the condition effect, $F(1, 42) = 3.534$, $p = 0.067$, nor the Condition × Time interaction, $F(1, 42) = 0.063$, $p = 0.804$, was significant. The period effect, $F(1, 42) = 2.815$, $p = 0.101$, and sequence effect, $F(1, 42) = 1.485$, $p = 0.230$, were also not significant. Thus, the conclusion that the temporal pattern of the sIgA response did not differ significantly between TSEP and HIIT was retained in the change-score sensitivity analysis. For CK, the condition effect remained significant, $F(1, 10) = 12.774$, $p = 0.005$, whereas the period effect, $F(1, 10) = 0.153$, $p = 0.704$, and sequence effect, $F(1, 10) = 2.390$, $p = 0.153$, were not significant. This sensitivity analysis supported the robustness of the greater CK increase following HIIT.

Collectively, the absence of significant baseline period or sequence effects and the absence of significant Period × Sequence interactions for most outcomes were broadly compatible with the adequacy of the 7-day washout period. Nevertheless, the significant Period × Sequence interactions for baseline sIgA and CK indicate that residual sequence-dependent baseline effects cannot be definitively excluded for these biomarkers. These exploratory findings should be interpreted cautiously because only six participants were included in each sequence.

Supplementary Table S2. Estimated within-condition changes and between-condition differences with 95% confidence intervals

Panel A. Acute salivary biomarkers

Outcome	Comparison	Estimated difference (95% CI)	Adjusted *p*
sAA ($U·L^{-1}$)	TSEP: Post − Pre	207 (138 to 276)	<0.001
	TSEP: Post-30 min − Pre	114 (45 to 183)	0.001
	TSEP: Post-30 min − Post	−93 (−162 to −24)	0.005
	HIIT: Post − Pre	224 (155 to 293)	<0.001
	HIIT: Post-30 min − Pre	146 (78 to 215)	<0.001
	HIIT: Post-30 min − Post	−77 (−146 to −8)	0.023
	TSEP − HIIT at Pre	51 (−41 to 143)	0.261
	TSEP − HIIT at Post	34 (−58 to 126)	0.450
	TSEP − HIIT at Post-30 min	18 (−74 to 110)	0.685
sIgA ($μg·mL^{-1}$)	TSEP: Post − Pre	−1.277 (−1.730 to −0.824)	<0.001
	TSEP: Post-30 min − Pre	−0.402 (−0.855 to 0.051)	0.098
	TSEP: Post-30 min − Post	0.875 (0.422 to 1.328)	<0.001
	HIIT: Post − Pre	−1.600 (−2.053 to −1.147)	<0.001
	HIIT: Post-30 min − Pre	−0.649 (−1.102 to −0.196)	0.003
	HIIT: Post-30 min − Post	0.951 (0.498 to 1.404)	<0.001
	TSEP − HIIT at Pre	−0.327 (−0.695 to 0.040)	0.080
	TSEP − HIIT at Post	−0.004 (−0.372 to 0.363)	0.981
	TSEP − HIIT at Post-30 min	−0.080 (−0.448 to 0.288)	0.663

Panel B. Salivary creatine kinase

Outcome	Comparison	Estimated difference (95% CI)	Adjusted *p*
CK ($U·L^{-1}$)	TSEP: Post-24 h − Pre	245 (202 to 287)	<0.001
	HIIT: Post-24 h − Pre	311 (268 to 354)	<0.001
	TSEP − HIIT at Pre	−12 (−54 to 31)	0.581
	TSEP − HIIT at Post-24 h	−78 (−121 to −35)	0.001

Panel C. Single-task tennis-specific performance

Outcome	Comparison	Estimated probability difference, percentage points	Adjusted *p*
**Forehand accuracy**	TSEP: Post − Pre	−16.0	0.019
	HIIT: Post − Pre	−3.6	0.588
	TSEP − HIIT at Pre	1.8	0.777

	TSEP − HIIT at Post	−10.6	0.116
**Backhand accuracy**	TSEP: Post − Pre	−14.8	0.028
	HIIT: Post − Pre	−8.8	0.191
	TSEP − HIIT at Pre	−1.6	0.809
	TSEP − HIIT at Post	−7.7	0.246
**Serve accuracy**	TSEP: Post − Pre	−10.9	0.097
	HIIT: Post − Pre	−5.9	0.369
	TSEP − HIIT at Pre	−2.5	0.699
	TSEP − HIIT at Post	−7.5	0.247

Panel D. Cognitive and dual-task performance

Outcome	Comparison	Estimated probability difference, percentage points	Adjusted *p*
Single-task 2-back accuracy	TSEP: Post − Pre	−23.2	<0.001
	HIIT: Post − Pre	−13.6	0.012
	TSEP − HIIT at Pre	−2.3	0.660
	TSEP − HIIT at Post	−11.9	0.029
Dual-task forehand accuracy	TSEP: Post − Pre	−11.3	0.091
	HIIT: Post − Pre	−3.5	0.599
	TSEP − HIIT at Pre	2.6	0.691
	TSEP − HIIT at Post	−5.2	0.433
Dual-task backhand accuracy	TSEP: Post − Pre	−11.1	0.084
	HIIT: Post − Pre	−8.6	0.186
	TSEP − HIIT at Pre	−1.6	0.801
	TSEP − HIIT at Post	−4.2	0.502
Dual-task serve accuracy	TSEP: Post − Pre	−7.5	0.230
	HIIT: Post − Pre	−6.7	0.296
	TSEP − HIIT at Pre	−4.2	0.515

Outcome	Comparison	Estimated probability difference, percentage points	Adjusted *p*
Dual-task cognitive accuracy	TSEP − HIIT at Post	−5.0	0.418
	TSEP: Post − Pre	−12.2	0.028
	HIIT: Post − Pre	−18.1	0.001
	TSEP − HIIT at Pre	−0.5	0.922
	TSEP − HIIT at Post	5.4	0.312
Combined dual-task accuracy	TSEP: Post − Pre	−9.4	0.063
	HIIT: Post − Pre	−7.8	0.115
	TSEP − HIIT at Pre	3.9	0.441
	TSEP − HIIT at Post	2.3	0.629

Note. For salivary biomarker outcomes in Panels A and B, estimated differences and 95% confidence intervals were derived from the estimated marginal means of the linear mixed-effects models. For the binomial accuracy outcomes in Panels C and D, probability differences were derived from the estimated marginal means of generalized linear mixed models with a binomial distribution and logit link and are reported in percentage points. Within-condition changes are expressed as the later measurement minus the earlier measurement, whereas between-condition differences are expressed as TSEP minus HIIT. Pairwise comparisons for Panels A and B were adjusted using the Bonferroni method, whereas comparisons for Panels C and D were adjusted using the sequential Bonferroni method within each fitted model. No multiplicity adjustment was applied across the separate outcome models; therefore, the comparisons should be interpreted as exploratory. CI, confidence interval; TSEP, tennis-specific exercise protocol; HIIT, high-intensity interval training; sAA, salivary alpha-amylase; sIgA, secretory immunoglobulin A; CK, creatine kinase.

Supplementary Table S3. Descriptive changes in relative motor and cognitive dual-task costs following TSEP and HIIT.

Outcome	TSEP Pre	TSEP Post	HIIT Pre	HIIT Post
**Forehand DTC (%)**	17.99 ± 4.77	14.38 ± 19.17	19.25 ± 6.37	19.21 ± 13.15
**Backhand DTC (%)**	22.57 ± 10.43	13.49 ± 39.75	18.83 ± 8.12	24.24 ± 10.18†
**Serve DTC (%)**	23.79 ± 4.55	21.11 ± 17.18	20.84 ± 11.09	24.05 ± 14.01
**Cognitive DTC (%)**	12.25 ± 16.92	−12.63 ± 30.40	15.10 ± 20.73	25.74 ± 29.85

Note. Values are presented descriptively as mean ± SD. DTC was calculated individually as [(single-task accuracy − dual-task accuracy)/single-task accuracy] × 100. Positive values indicate poorer performance under dual-task conditions, whereas negative values indicate that dual-task accuracy exceeded the corresponding single-task accuracy. One backhand DTC value at HIIT Post could not be calculated because the corresponding single-task accuracy was zero; therefore, n = 11 for this cell and n = 12 for all other cells. DTC outcomes were not subjected to inferential testing because some ratio-derived scores showed substantial departures from normality and denominator instability. Accordingly, these findings should be interpreted as exploratory. TSEP, tennis-specific exercise protocol; HIIT, high-intensity interval training; DTC, dual-task cost; SD, standard deviation.

Motor and cognitive dual-task costs are presented descriptively in Supplementary Table S3. Positive values generally indicated poorer performance under dual-task conditions, although the magnitude and direction of DTC varied across conditions and time points. Because some ratio-derived DTC outcomes showed substantial departures from normality and one backhand DTC value was undefined owing to a zero single-task denominator, inferential analyses based directly on these scores were not considered sufficiently robust. Accordingly, the DTC findings are reported as exploratory descriptive outcomes and should be interpreted cautiously.

Table S4. Actual heart-rate responses during the TSEP and HIIT protocols

Variable	TSEP (Mean ± SD)	HIIT (Mean ± SD)
Mean HR (beats·min^{-1})	167.5 ± 1.8	181.7 ± 1.6
Peak HR (beats·min^{-1})	179.3 ± 2.0	190.6 ± 1.6
Mean HRR (%)	78.6 ± 1.0	90.3 ± 0.8
Peak HRR (%)	88.3 ± 1.2	97.6 ± 0.8

Table note: Values are mean ± SD (n = 12). HR, heart rate; HRR, heart rate reserve; TSEP, tennis-specific interval exercise protocol; HIIT, high-intensity interval training. HRmax was estimated using 208 − 0.7 × age, and HRR percentages were calculated using the Karvonen method.

Actual heart-rate responses during the exercise protocols are presented in Supplementary Table S4. Mean HR was 167.5 ± 1.8 beats·min^{-1} during TSEP and 181.7 ± 1.6 beats·min^{-1} during HIIT, corresponding to 78.6 ± 1.0% and 90.3 ± 0.8% of HRR, respectively. Peak HRR reached 88.3 ± 1.2% during TSEP and 97.6 ± 0.8% during HIIT, indicating that participants approached the prescribed high-intensity targets, particularly during HIIT.
